# Treatment May Be Harmful: Mechanisms/Prediction/Prevention of Drug-Induced DNA Damage and Repair in Multiple Myeloma

**DOI:** 10.3389/fgene.2019.00861

**Published:** 2019-09-18

**Authors:** Claire Gourzones, Caroline Bret, Jerome Moreaux

**Affiliations:** ^1^IGH, CNRS, Univ Montpellier, France; ^2^Department of Biological Hematology, CHU Montpellier, Montpellier, France; ^3^Univ Montpellier, UFR de Médecine, Montpellier, France; ^4^Institut Universitaire de France, Paris, France

**Keywords:** multiple myeloma, DNA damage, genomic instability, drug resistance, genotoxic agents

## Abstract

Multiple myeloma (MM) is a malignancy characterized by accumulation of malignant plasma cells within the bone marrow (BM). MM is considered mostly without definitive treatment because of the inability of standard of care therapies to overcome drug-resistant relapse. Genotoxic agents are used in the treatment of MM and exploit the fact that DNA double-strand breaks are highly cytotoxic for cancer cells. However, their mutagenic effects are well-established and described. According to these effects, chemotherapy could cause harmful DNA damage associated with new driver genomic abnormalities providing selective advantage, drug resistance, and higher relapse risk. Several mechanisms associated with MM cell (MMC) resistance to genotoxic agents have been described, underlining MM heterogeneity. The understanding of these mechanisms provides several therapeutic strategies to overcome drug resistance and limit mutagenic effects of treatment in MM. According to this heterogeneity, adopting precision medicine into clinical practice, with the development of biomarkers, has the potential to improve MM disease management and treatment.

## Introduction

Multiple myeloma (MM) is the second most common hematological malignancy, with an incidence ranging from 4 to 6 per 100,000/year in the United States (Braggio et al., 2015). Malignant plasma cells display prominent genomic abnormalities arising during tumorigenesis and accumulating during disease progression. This series of complex molecular events involved in MM development includes chromosomal abnormalities, oncogene activation, and cellular communication signals dysregulation. MM treatment strategies have significantly evolved during the last decades with an expanding arsenal of anti-MM therapies. This was associated with a three-fold improvement in median survival. According to these significant advances, long-term side effects and harmful effects of treatment are an important issue with the development of drug-resistant subclones and formation of therapy-related cancers caused by lesions they create in normal cells. Thus, understanding the DNA damages induced by anti-MM therapies and DNA damage response is important to improve patient survival and reduce harmful effects related to treatment.

## Genomic Instability in Multiple Myeloma

Genome integrity is constantly assailed by diverse arrays of insults. These include genotoxic agents of exogenous origin and endogenous sources of DNA damage, such as reactive oxygen species, collisions between DNA replication and transcription, and programmed genomic rearrangements (Aguilera and Gomez-Gonzalez, 2008). B cells are particularly challenged in this regard. During their maturation, B lymphocytes are subjected different to genetic alterations, including V, D and J immunoglobulin genes recombination, Ig class switch recombination (CSR), and somatic hypermutation (SHM). To prevent immune deficiency, autoimmunity, or cancer, these biological processes should be tightly regulated (Gennery et al., 2000). MM cells are characterized by genomic instability, including chromosomal instability, mutations, and microsatellite instability. Recent genome sequencing studies of MM cells have uncovered the major genomic instability and molecular and subclonal heterogeneity of the disease (Lohr et al., 2014; Vikova et al., 2019).

## DNA-Damaging Agents Used in Multiple Myeloma Treatment

Melphalan DNA-damaging agent was introduced for the treatment of MM in 1962. Several combinations have been developed to the improve outcome of patients, including other DNA-damaging agents (**Table 1**). The high-dose melphalan regimen combined with autologous hematopoietic stem cell transplant (ASCT) has long been considered frontline therapy for newly diagnosed patients. Melphalan is a nitrogen mustard known to induce mono-alkylation of adenine and guanine together with interstrand DNA crosslinks (Osborne et al., 1995). Monoadducts represent 95% of the lesions, whereas 5% are interstrand DNA crosslinks (ICLs) (Muniandy et al., 2010). In MM cells, ICL number is correlated with the melphalan concentration used (Spanswick et al., 2002). ICLs are highly toxic for MM cells.

**Table 1:** DNA-damaging chemotherapeutic drugs used in MM.

Agents
Melphalan (Alkeran^®^)
Cyclophosphamide (Cytoxan^®^)
Doxorubicin (Adriamycin^®^)
Busulfan (Myleran^®^)
Vincristine (Oncovin^®^)
VP-16 (Etoposide^®^)
Bendamustine (Treanda^®^)
Pegylated liposomal doxorubicin (Doxil^®^)
Melphalan flufenamide hydrochloride (Melflufen^®^)
Cisplatin (Platinol^®^)

Results from studies with a long-term follow-up of patients treated with high-dose chemotherapy and ASCT demonstrated that 10%–15% of the patients remain alive after more than 10 years without relapse (Barlogie et al., 2006; van de Velde et al., 2007; Martinez-Lopez et al., 2011), demonstrating the efficacy of this strategy in MM (van Rhee et al., 2014). The novel drugs such as immunomodulatory agents (IMiDs) and proteasome inhibitors (PIs) used during induction, consolidation, and maintenance stages have significantly improved the outcome of the patients (Barlogie et al., 2006; Cavo et al., 2010; Ladetto et al., 2010; Moreau et al., 2011; Neben et al., 2012; Sonneveld et al., 2012).

Other genotoxic drugs are used to treat patients with MM, including cyclophosphamide, doxorubicin, busulfan, vincristine, etoposide, cisplatin, bendamustine, lyposomal doxorubicin, and melphalan flufenamide hydrochloride. Use of chemotherapeutic agents without cross-resistance could enhance peripheral blood stem cell collection and improve patient outcome related to better antitumor efficacy before ASCT. Cyclophosphamide is an alkylating agent inducing ICLs. Doxorubicin induces DNA double-strand DNA breaks (DSBs) related to intercalation into DNA and inhibition of topoisomerase II (TopII). Doxorubicin can also induce DNA adducts, free radicals release, and formaldehyde-dependent ICL formation (Bret et al., 2013). Etoposide is a DNA-damaging agent that induces DNA damage and inhibits DNA replication by suppressing the relaxation activity of TopIIA topoisomerase (Dobbelstein and Sorensen, 2015). Busulfan is a bifunctional alkylating agent. Vincristine is a microtubule-targeting agent that was demonstrated to increase DNA damage induced by DNA-damaging agents (Poruchynsky et al., 2015). Cisplatin is a platinum compound that modifies DNA, leading to the formation of intrastrand or interstrand crosslinks between bases. Cisplatin is used in DCEP regimen in combination with dexamethasone, etoposide, and cyclophosphamide (Dadacaridou et al., 2007; Park et al., 2014). Pegylated liposomal doxorubicin is a formulation of doxorubicin in liposomes with a prolonged circulation time (Voorhees et al., 2015). Bendamustine combines bifunctional alkylating nitrogen mustard group and a purine-like benzamidazol nucleus and was shown to overcome melphalan resistance in human myeloma cell lines (Cives et al., 2013). Melphalan flufenamide hydrochloride is a lipophilic alkylator characterized by intracellular hydrolysis after cellular uptake and release of active melphalan (Ray et al., 2016).

## Drug Resistance Mechanisms to DNA-Damaging Agents

### Drug Efflux, Cell Communication Signals, and Drug Metabolism

**Figure 1:**
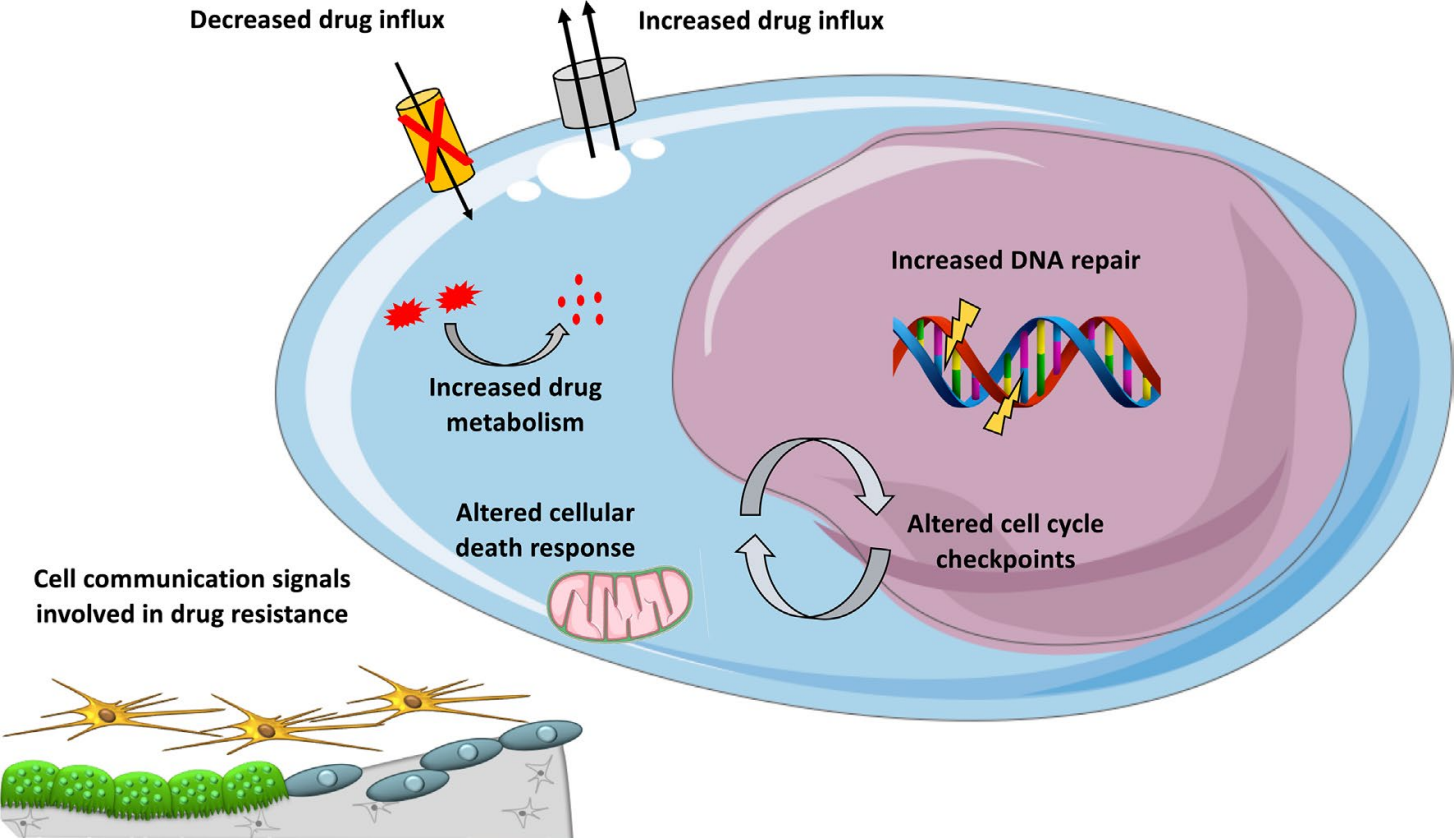
Mechanisms involved in DNA-damaging drug resistance in MM. Overview of mechanisms contributing to resistance to DNA-damaging agents in MM, including cellular extrusion of the drugs by ATP-dependent pumps, decreased drug influx, increased drug inactivation by metabolism, inactivation of apoptotic pathways, enhanced DNA repair, and altered cell cycle checkpoints and cell communication signals provided by the microenvironment.

An important mechanism of multidrug resistance in cancer is the overexpression of ATP-binding cassette (ABC) transporters (Kathawala et al., 2015) (**Figure 1**). This family of transmembrane proteins use the energy generated by ATP hydrolysis to efflux cytotoxic compounds. The most studied ABC is dependent efflux pump P-glycoprotein (P-gp) coded by the *MDR1/ABCB1* gene. In MM, no significant expression of P-gp was detected in newly diagnosed MM and in patients treated with melphalan (Grogan et al., 1993). P-gp overexpression was demonstrated to be associated with resistance to glucocorticoid, etoposide, doxorubicin, and vincristine (Dalton, 1997). VAD treatment (vincristine, doxorubicin, and dexamethasone) was associated with P-gp overexpression in MM patients (Sonneveld et al., 2001; Yang et al., 2003). However, a clinical trial with ABCB1 inhibitor (Zosuquidar) did not show any benefit in progression-free or overall survival in refractory MM patients when combined with vincristine, doxorubicin, and dexamethasone (Friedenberg et al., 2006).

The behavior of MM cells is determined not only by their genetic or epigenetic background but also by their BM microenvironment. The majority of myeloma growth factors (MGFs) is secreted by the BM environment compared to autocrine MGFs (Mahtouk et al., 2010). Several studies have provided a comprehensive overview of MGF expression in the different BM cell subpopulations of MM patients (Podar et al., 2009; Mahtouk et al., 2010). Interactions between MM cells and bone marrow microenvironment could also play a role in DNA-damaging agents drug resistance (**Figure 1**). We have documented the rise of large concentrations of IL-6 9 days after high-dose melphalan in patients (Condomines et al., 2010). This large concentration of IL-6 will facilitate melphalan-resistant MMCs to survive within the BM. Patients treated with high-dose melphalan, stem cell transplantation, and anti-IL-6 antibody had a survival advantage when mixed with a large cohort of matched patients treated with melphalan and stem cell transplantation alone (Rossi et al., 2005). Cell adhesion-mediated drug resistance to doxorubicin, vincristine, and melphalan was described using human myeloma cell lines and primary MM cells from patients (Damiano et al., 1999; Noborio-Hatano et al., 2009; Neri et al., 2011a; Di Marzo et al., 2016). Bortezomib could overcome cell adhesion-mediated drug resistance through VLA-4 downregulation and inhibition of MM cell adhesion to stroma (Noborio-Hatano et al., 2009; Neri et al., 2011a). Cell adhesion-mediated drug resistance could also protect MM cells from etoposide toxicity (Hazlehurst et al., 2000). Targeting cell-to-cell communication between MM cells and BM microenvironment could improve current therapeutic strategies using DNA-damaging agents.

*In vivo*, drugs could be metabolized (**Figure 1**) through a number of reactions occurring through two distinct consecutive phases: phase I and phase II drug metabolism. Phase I drug metabolizing enzymes consist primarily of oxidases, reductases, and dehydrogenases, and phase II enzymes play an important role in biotransformation and inactivation of drugs. A study reported that MMCs of patients with a favorable outcome after treatment with high-dose therapy and ASCT are characterized by an overexpression of genes coding for xenobiotic receptors and their downstream targets, including phase I and phase II drug metabolism enzymes and transporters (Hassen et al., 2014). At the opposite, high-risk patients were characterized by overexpression of genes involved in *Nrf2* and *ARNT* pathways (Hassen et al., 2014). These data underline a role of drug metabolism in chemotherapy resistance in MM and suggest that inhibitors targeting these pathways could open new perspectives to alleviate or overcome drug resistance.

### DNA-Damaging Agents and DNA Repair Pathways

The fact that DNA double-strand breaks are highly cytotoxic is exploited by DNA-damaging agents used in the treatment of MM. According to the type of DNA damage, specific DNA repair pathways will be used to cope with DNA insults. For nucleotide lesions occurring on single strands, base excision repair (BER), nucleotide excision repair (NER), and mismatch repair (MMR) will be involved. For DSBs, there are two major pathways, including nonhomologous end-joining (NHEJ) and homologous recombination (HR) DNA repair. The DNA damage response (DDR) sensor proteins will be involved in the detection of damaged DNA, leading to cellular response activation, including one or more DNA repair pathways. For DSBs, Ku proteins and MRN complex are the predominant sensors. Fanconi anemia proteins, Poly (ADP -ribose) polymerase (PARP), mismatch repair proteins (including MSH2, MSH3, MSH6, PMS2, and MLH1), and NER proteins (including XPC, CSA, and DDB2) are other DNA-damage sensors (Brown et al., 2017).

#### Single-Strand Damage DNA Repair

##### Nucleotide Excision Repair

**Figure 2:**
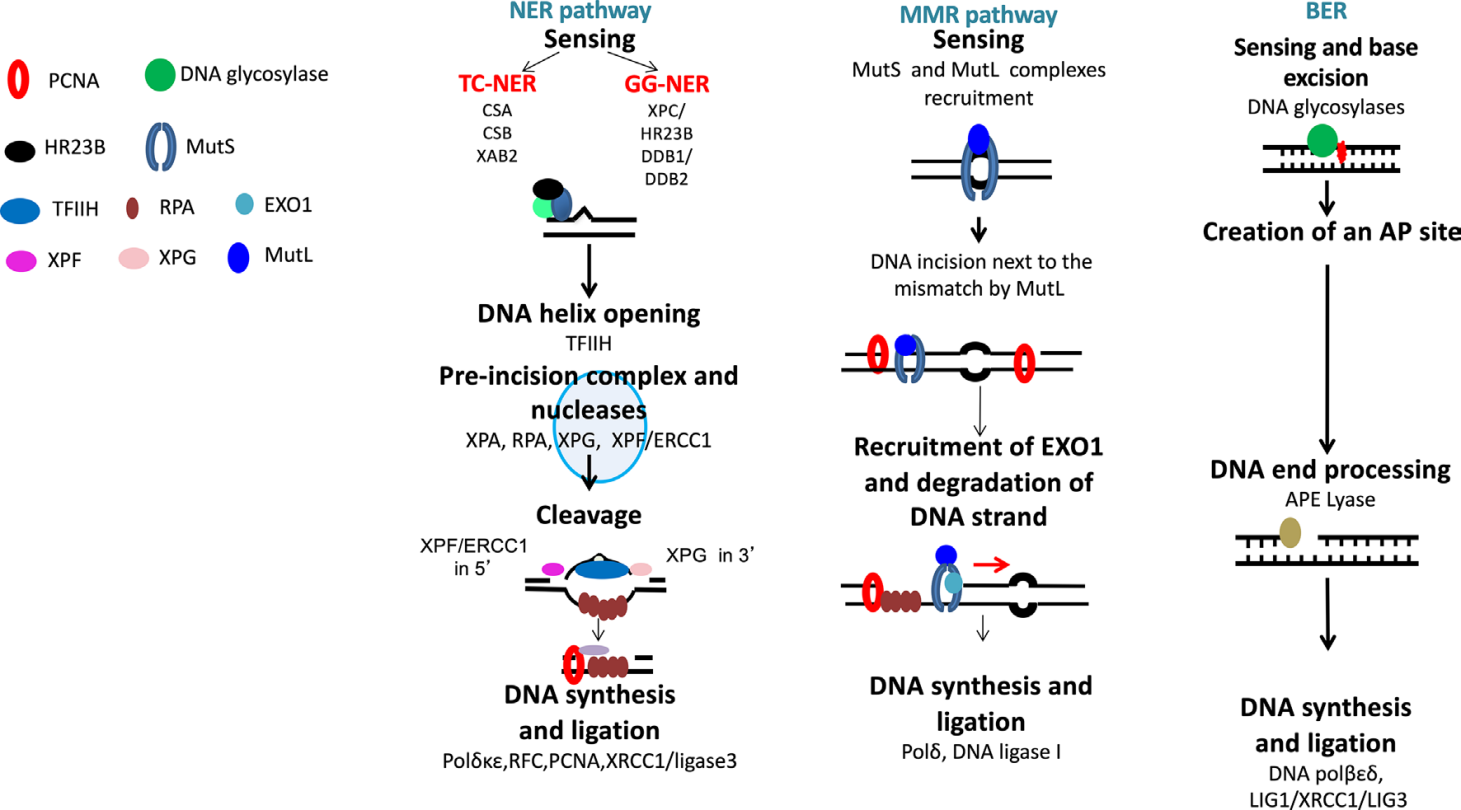
DNA single-strand damage repair. Base damages are repaired by base excision repair, bulky adducts by nucleotide excision repair, and base mismatch by mismatch repair pathway.

**Figure 3:**
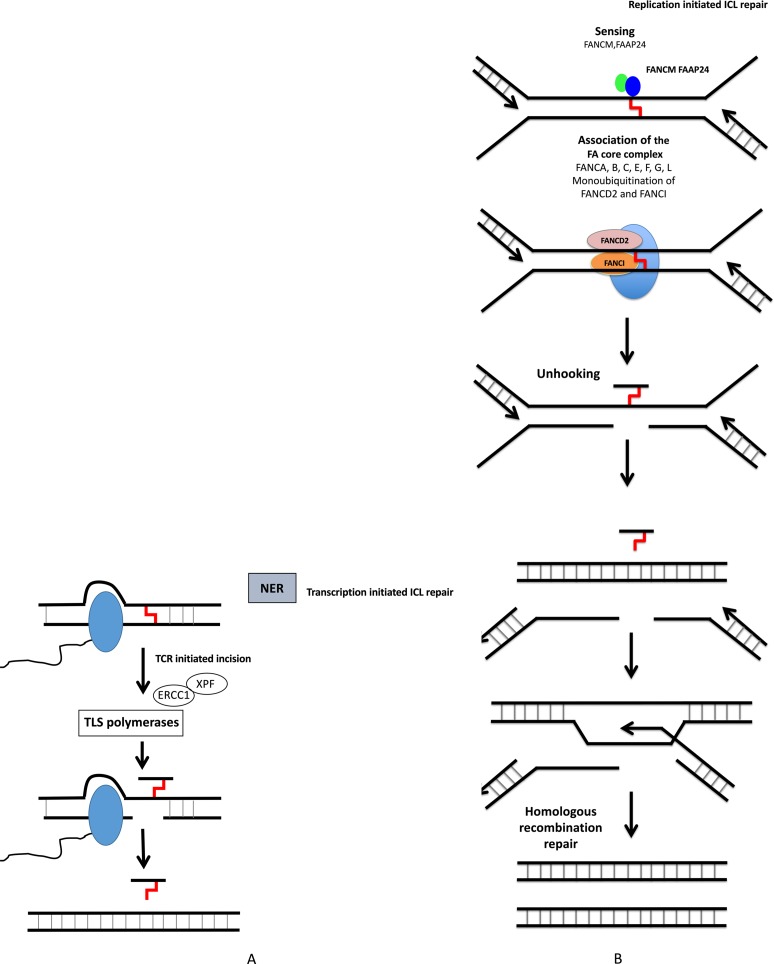
DNA interstrand crosslink repair. ICL repair can be initiated either at a stalled RNA polymerase **(A)** or at a replication fork **(B)**. **(A)** ICLs in DNA will stall RNA polymerase during transcription. The RNA polymerase will either backtrack or be degraded during subsequent repair involving translesion (TLS) polymerases and NER pathway. **(B)** The removal of ICL during S and G2 phases involves the Fanconi anemia pathway, with sensing of ICL by FANCM, and then recruitment of protein complex, resulting in ICL removal, creation of DSB, which is repaired by homologous recombination.

Nucleotide excision repair (NER) removes helix-distorting adducts on DNA that could be caused by UV or radiation and participates in the repair of ICLs44 (**Figures 2** and **3A**). NER can be coupled to transcription [transcription-coupled nucleotide excision repair (TC-NER)] opposed to global genome nucleotide excision repair (GG-NER) (Friedberg, 2001; Hanawalt and Spivak, 2008). In cancer cells exposed to genotoxic agents, NER plays a key role in removal and repair of the DNA damage (Kirschner and Melton, 2010). It has been demonstrated that NER is a major DNA repair mechanism that removes cisplatin-induced DNA damage. In solid tumors, high expression of ERCC1, involved in NER, is associated with resistance to platinum-based therapy. NER removes helix-distorting adducts participating in the ICL repair. ERCC1 and XP proteins play major roles in the repair of DNA adducts and ICLs (**Figure 3A**). Furthermore, deficiency in NER was associated with higher sensitivity to platinum agents and a reduced capacity to repair ICLs (Kirschner and Melton, 2010).

##### Base Excision Repair

The base excision repair (BER) pathway repairs DNA damaged bases (Lindahl, 1993) (**Figure 2**). Conventional BER is initiated by specific DNA glycosylases that will hydrolyze N-glycosylic bond between the damaged base and the sugar phosphate backbone. This process is followed by end processing mechanism, repair synthesis, and ligation (Caldecott, 2008). However, different mechanisms could participate in BER pathways, depending on the physiological state of cancer cells and the type of DNA glycosylase involved (Caldecott, 2008).

##### Mismatch DNA Repair

Mismatch DNA repair (MMR) is involved in the repair of replication errors associated with nucleotide deletion or insertion or causing incorporation of a wrong nucleotide (**Figure 2**). MSH2-MSH6 defects in MMR are associated with drug resistance to temozolomide, nucleoside analogs, and platinum agents in solid cancers and Acute myeloid leukemia (AML) (Karran and Bignami, 1994; Fordham et al., 2011).

#### DSBs DNA Repair

**Figure 4:**
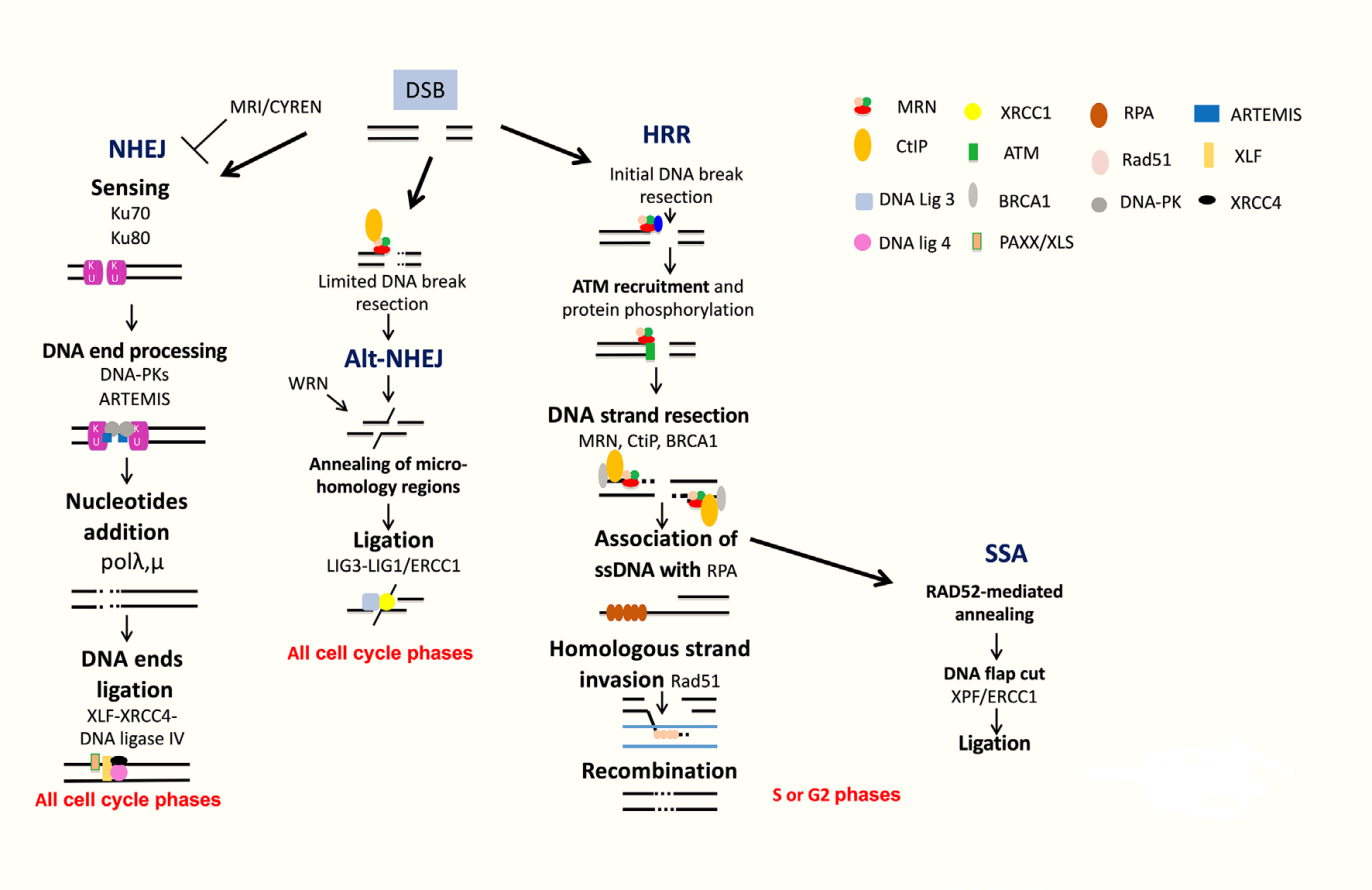
DNA double strand break repair. DSB are repaired by NHEJ or HRR pathways. NHEJ initiates with broken ends bound by Ku, which protects ends, leading to repair with partial resection and ligation of DNA ends. Alt-NHEJ is an alternative less accurate pathway. HRR is an accurate pathway. The DNA ends of the lesion are resected to allow invasion of the single strand into the sister chromatid used as a template for precise resynthesis of damaged DNA part. SSA is used only when two homologous regions flank the DSB site and is inaccurate.

DSBs can be generated during ICLs repair, by ROS, by DNA damaging used in MM treatment and during DNA replication when cells progress in S phase before completing single-strand DNA damage repair (Ciccia and Elledge, 2010; Deans and West, 2011). Several partially independent sensors can detect DSBs, including Ku70/Ku80, PARP, MRN, and RPA. Homologous recombination operates in the S and G2 phases of the cell cycle. Homologous recombination pathway is conservative and requires a homologous DNA template (**Figure 4**). At the opposite, NHEJ is nonconservative with direct ligation of DSB ends (**Figure 4**).

##### Homologous Recombination DNA Repair

The homologous recombination DNA repair (HRR) pathway is complex and occurred during S and G2 phases of the cell cycle (Ciccia and Elledge, 2010; Deans and West, 2011; Gourzones-Dmitriev et al., 2013). The DNA ends of the lesion are resected to allow invasion of the single strand into the sister chromatid used as a template for precise resynthesis of damaged DNA part. HRR plays an important role in genomic stability (Curtin, 2012; Curtin, 2013). The MRN complex (MRE11-RAD50-NBS1) recognizes DSBs, and CtIP recruits RPA (Williams et al., 2007; Jasin and Rothstein, 2013). BRCA1 interacts also with MRN and CtIP and promotes HRR and single-strand annealing (SSA). The resulting 3′ ssDNA tails are bound by RPA, which is replaced with Rad51. The reaction is mediated by Rad52, Rad55, and Rad57. The RAD51 nucleoprotein filament promotes homology search. This is followed by exchange strand between the intact sister chromatid and the broken duplex and strand extension by DNA polymerase (**Figure 4**). HRR repairs DSBs, which occurs through exposure to topoisomerase inhibitors and DNA crosslinking agents used in MM treatment, including etoposide and doxorubicin. HRR plays a major role in stalled replication fork restart and ICL repair in association with the Fanconi anemia (FA) pathway (**Figure 3B**). HRR is more active in MM cells compared to normal plasma cells (Shammas et al., 2009; Roddam et al., 2010). Higher expressions of RAD50 and RAD51 proteins involved in HRR have been reported in primary MM cells and MM cell lines compared to normal plasma cells in association with increased HRR activity (Shammas et al., 2009; Roddam et al., 2010). The proliferation assessment based on propidium iodide incorporation (plasma cell labeling index) (San Miguel et al., 1995) or the assessment of Ki-67-expressing cells of malignant plasma cells (Alexandrakis et al., 2004) has been shown to be a powerful and independent predictor of survival in MM. (Greipp et al., 1988) The role of HRR in drug resistance of the small fraction of proliferating MM cells may be of particular importance in MM.

##### Single-Strand Annealing

SSA is used only when two homologous regions flank the DSB site. In this context, the homologous regions are exposed, and after annealing and cleavage of the DNA overhang, ligation of the ends results in the deletion of the intervening region (**Figure 4**). According to this process, SSA is inaccurate. Translocations induced by SSA have been reported when DSBs are located in repeats on different chromosomes (Hartlerode and Scully, 2009; Ciccia and Elledge, 2010).

##### NHEJ DNA Repair

NHEJ does not use significant homology at the broken ends. DSBs are sensed by Ku70/Ku80 that bind and activate DNA-PKcs protein kinase, leading to the recruitment and activation of DNA end-processing enzymes, such as ARTEMIS, template-independent polymerases (polymerases λ and μ), and XLF-XRCC4-DNA ligase 4 complex (**Figure 4**). More recently, several new accessory NHEJ factors, including PAXX/XLS and CYREN/MRI, have been described. These factors have been reported to be required for DNA ligation. PAXX/XLS is an accessory related to NHEJ pathway and presenting overlapping functions with XLF (Tadi et al., 2016). CYREN is a cell cycle-dependent inhibitor of NHEJ pathway, promoting repair by HR pathway (Arnoult et al., 2017; Hung et al., 2018). A correlation between polymorphisms or aberrant expressions of *XRCC4, XRCC5* (encoding Ku80), *XRCC6* (encoding Ku70), *ARTEMIS*, and *LIG4* (encoding DNA ligase 4) genes and risk of MM development has been reported (Roddam et al., 2002; Hayden et al., 2007; Roddam et al., 2010). Interestingly, high expression of *XRCC5* and *ARTEMIS* genes was associated with a poor prognosis in MM patients (Calimeri et al., 2012). A recent study investigated the functionality of DSB repair in MM cells and identified upregulation of ARTEMIS, DNA-PKcs, and XRCC4 proteins in MM cells (Herrero et al., 2015). NHEJ activity was also significantly elevated in MM cells compared to a normal lymphoblastoid cell line (Herrero et al., 2015). Furthermore, MMSET was recently identified to be involved in DNA damage response as a sensor (Hajdu et al., 2011). MMSET, like RIF1, is an ATM-dependent DDR factor. MMSET accumulates at DSBs, leading to recruitment of 53BP1. Recruitment of 53BP1 to DSBs is dependent on histone H4 methylation by MMSET (Pei et al., 2011). However, two other groups demonstrated that MMSET had no effect on 53BP1 foci formation and H4K20 methylation (Hsiao and Mizzen, 2013; Tuzon et al., 2014). During B-cell development, MMSET is also involved in class-switch recombination accumulating at immunoglobulin gene-switch regions with H3K36me2 and γH2AX. Furthermore, MMSET depletion led to defects in class-switch recombination (Pei et al., 2013). DSBs generated during CSR modifications of B-lymphocyte Ig genes are mainly repaired by NHEJ (Lieber, 2010). H3K36 methylation, induced by MMSET and other histone methyltransferases, can also influence DNA repair pathway choice (Fnu et al., 2011; Aymard et al., 2014; Carvalho et al., 2014; Pfister et al., 2014). More recently, it was shown that MMSET is necessary for efficient NHEJ and HRR (Shah et al., 2016). MMSET depletion resulted in decreased expression of DNA repair genes and significant reduction in the recruitment of DNA repair proteins at DNA damage sites. MMSET overexpression is associated with increased DNA repair efficiency. Loss of MMSET combined with melphalan treatment leads to decreased tumor mass and increased survival in mice (Shah et al., 2016). MMSET-overexpressing cells demonstrate increased γH2AX after treatment with DNA-damaging agents, suggesting a role in drug resistance through accelerated DNA repair (Shah et al., 2016). Given the association of t(4;14), involving MMSET, with poor outcome in MM, this potential perturbation of DNA repair may prove highly relevant to disease progression and drug resistance. More recently, Wang and Goldstein (2016) demonstrated that Dicer- and Drosha-dependent diRNAs play a role in guiding molecules to promote the recruitment of MMSET and other proteins to DSB sites. Several groups have demonstrated that H3K36 is the main target of MMSET without effect on H4K20 (Li et al., 2009; Martinez-Garcia et al., 2010; Popovic et al., 2014). Several studies reported that H3K36 mark favors NHEJ as DNA repair pathway choice (Fnu et al., 2011; Aymard et al., 2014; Carvalho et al., 2014; Pai et al., 2014; Pfister et al., 2014). H3K36me2 increase and H3K27me3 decrease in MMSET-overexpressing cells are associated with a more open chromatin state (Martinez-Garcia et al., 2010; Popovic et al., 2014). This chromatin state is associated with an increased DNA damage when cells are treated with a DNA-damaging agent (Shah et al., 2016). However, DNA repair proteins could access more easily to chromatin and facilitate efficient DNA repair (Ransom et al., 2010; Soria et al., 2012), as observed in cells with high MMSET expressions (Shah et al., 2016).

##### Alternative NHEJ DNA Repair

Alt-NHEJ is an alternative end-joining pathway that is detected when the classical NHEJ pathway is impaired (Nussenzweig and Nussenzweig, 2007; Mladenov and Iliakis, 2011) (**Figure 4**). Alt-NHEJ is less accurate, requires more extensive end resection, and frequently uses microhomology. DNA ends are resected up to regions of microhomology, annealed, and ligated by XRCC1/ligases 1 and 3 (Ciccia and Elledge, 2010; Shaheen et al., 2011; Simsek et al., 2011; Boboila et al., 2012). The 53BP1 protein protects DNA ends from nucleolytic degradation and thereby prevents microhomology mediated repair (Bothmer et al., 2010). Moreover, it has been implicated in the chromosomal translocation process involved in lymphoid tumorigenesis (Nussenzweig and Nussenzweig, 2007; Kotnis et al., 2009). Overexpression of proteins involved in alt-NHEJ, including DNA ligase 3, was also reported in MM cells (Herrero et al., 2015). Activity of this highly mutagenic pathway was identified in MM cells through detection of largest deletions and higher sequence microhomology at DNA lesion sites and could be decreased by alt-NHEJ inhibition (Herrero et al., 2015).

## DNA Repair and Resistance to DNA-Damaging Agents in Multiple Myeloma

Cyclophosphamide is a genotoxic agent inducing ICLs. Melphalan is also a nitrogen mustard that induces ICLs. The vast majority of the lesions induced by melphalan are monoadducts and 5% ICLs (Osborne et al., 1995; Muniandy et al., 2010). The ICL number induced by melphalan in MM cells is correlated with the concentration used (Spanswick et al., 2002). Doxorubicin induces DNA adducts, DSB, free radicals release, and formaldehyde-dependent ICL formation. A study reported that MM cells from patients previously treated by melphalan are able to repair ICLs *in vitro*, whereas MM cells from untreated patients could not (Spanswick et al., 2002). Furthermore, melphalan-induced DNA damage, *in vitro*, in peripheral blood mononuclear cells is a predictor for clinical outcome in patients treated by high-dose melphalan and ASCT (Dimopoulos et al., 2007). A significant association between polymorphisms of genes involved in DNA repair and melphalan resistance was identified in MM (Dumontet et al., 2010). In a cohort of MM patients treated with high-dose melphalan and ASCT, polymorphisms of *PARP, RAD51, PCNA, OGG1, XPC, BRCA1, ERCC1, BARD1*, and *TP53BP1* are associated with the outcome and overall survival of patients (Dumontet et al., 2010). These genes are significantly enriched in genes involved in HRR and NER necessary for ICL repair. NER removes helix-distorting adducts on DNA and contributes to the repair of ICLs. Furthermore, reduced capacity to repair ICL has been associated with NER deficiency (**Figure 3A**) together with higher sensitivity to platinum agents (Kirschner and Melton, 2010) ERCC2 and XRCC3 gene polymorphisms are associated with treatment outcome and drug resistance in patients treated with high-dose melphalan and ASCT (Vangsted et al., 2007). In MM cell lines resistant to melphalan, overexpression or *BRCA1*, *BRCA2*, *FANCA*, *FANCC*, *FANCF*, *FANCL*, and *RAD51C*; upregulation of HRR; and Fanconi pathways were identified. Depletion of FANCF could overcome melphalan resistance, and FANCF overexpression protects MM cells from melphalan toxicity (Chen et al., 2005). FA pathway appears as a key player in DNA-damaging agent resistance. NF-κB, constitutively activated in MM (Demchenko and Kuehl, 2010; Chapman et al., 2011), was shown to transcriptionally regulate FA pathway genes (Yarde et al., 2009). According to that, proteasome inhibitors affect DNA repair pathways in MM through several mechanisms. Proteasome inhibitors block NF-kB-mediated transcriptional activation of FA genes (Yarde et al., 2009). PIs also inhibit the decrease in FANCD2 monoubiquitination and the expression of FANC proteins (Jacquemont and Taniguchi, 2007). PIs also target HRR by blocking NBS1, BRCA1, phospho-ATM, and Rad51 recruitment (Murakawa et al., 2007). PI treatment reduces the pool of available nuclear ubiquitin and impede FANCD2 and H2AX ubiquination (Neri et al., 2011b). Bortezomib treatment may prevent DNA resection through inhibiting proteasomal degradation of proteins involved in chromatin relaxation (Jacquemont and Taniguchi, 2007; Murakawa et al., 2007). This mechanism impedes the recruitment of RPA onto ssDNA (Jacquemont and Taniguchi, 2007; Murakawa et al., 2007). This effect of PIs on DNA repair could explain the synergistic activity of PI treatment with high-dose melphalan before ASCT (Roussel et al., 2010). MMSET was also involved in melphalan drug resistance through significant increases of DNA repair efficiency (Shah et al., 2016). Several studies demonstrated a role of BER in DNA-damaging agent resistance in MM. APE1 depletion was reported to sensitize MM cells to melphalan treatment (McNeill et al., 2009). More recently, gene expression profile analyses revealed a prognostic value of genes involved in FA (*RMI1, FANCI*, and *FANCA*), NER (*PCNA, RPA3, LIG3, POLD3, ERCC4, POMD1, ERCC1*, and *ERCC5*), NHEJ [*WHSC1* (MMSET), *RIF1, XRCC5* (Ku80), *PNKP*, and *POLL*], MMR (*EXO1* and *MSH2*), and HRR (*EXO1, BLM, RPA3, RAD51, MRE11A*, and *ATM*) pathways in MM (Kassambara et al., 2014). Based on these prognostic genes, NHEJ, HRR, FA, and NER gene-based risk scores were created, allowing identification of high-risk MM patients in two independent cohorts of patients treated with high-dose melphalan and ASCT (Kassambara et al., 2014). Furthermore, a DNA repair risk score, incorporating all of the 22 DNA repair prognostic genes presented a strong prognostic value for both event-free survival and overall survival in two independent cohorts of MM patients. This gene-based DNA repair score remained an independent prognostic factor when tested together with known prognostic factors, including previously published gene expression profiling (GEP)-based risk scores, t(4;14), del17p and with standard clinical prognostic factors, ISS, b2m, and albumin. These DNA repair scores represent powerful tools to develop synthetic lethality approaches and exploit the addiction of MM cells to a specific DNA repair pathway (Kassambara et al., 2014).

RECQ helicases playing a role in repair of damaged replication forks, DNA damage response, and homologous recombination are also involved in cancer cell drug resistance (Futami et al., 2008a; Futami et al., 2008b; Arai et al., 2011; Viziteu et al., 2016). In MM, high RECQ1 expression is associated with resistance to genotoxic agents and poor prognostic value in several independent cohorts of MM patients (Viziteu et al., 2017). MM cells overexpressing RECQ1 are able to repair DNA breaks induced by genotoxic agents more efficiently, conferring drug resistance (Viziteu et al., 2017). Interestingly, the abnormal overexpression of RECQ1 in MM is linked to aberrant methylation of miR-203. DNA methylation inhibitor treatment induces upregulation of miR-203 followed by RECQ1 downregulation and hypersensitivity to treatments (Viziteu et al., 2017).

In MM patients characterized by 1q21 amplification, high expression of ILF2 has also been reported to participate in resistance to genotoxic agents through regulation of DNA repair gene splicing transcription (Marchesini et al., 2017).

## Mutagenic Effects of Chemotherapy in Multiple Myeloma

Genotoxic agents can induce mutagenic effects. DNA alkylating agents and intercalating agents have been associated with mutations signatures (Szikriszt et al., 2016). According to these effects, chemotherapy could cause harmful DNA damage associated with new driver genomic abnormalities, providing selective advantage, drug resistance, and higher relapse risk (Weinhold et al., 2016). Alkylating agents also modify the redox potential of cancer cells. This mechanism has been reported for melphalan and bendamustine that increase the level of reactive oxygen species (ROS), inhibit the activity of thioredoxin reductase, and activate p53 pathway (Witte et al., 2005; Surget et al., 2014). Interestingly, reduced glutathione (GSH) protects MM cells from melphalan-induced toxicity without affecting the ability of melphalan to induce DNA damage (Gourzones et al., 2019). Deregulation of genes involved in response to oxidative stress is associated with a poor outcome and Melphalan resistance in MM (Gourzones et al., 2019). The DNA damage accumulation resulting from survival advantage mediated by the redox system upregulation could participate in new genomic driver events and poor outcome in MM (Gourzones et al., 2019). In this context, the selective pressure of chemotherapy may result in new driver mutations associated with MM cell subclonal selection and drug resistance, as illustrated with double hit events involving tumor suppressor genes, including *TP53* and *FAM46C* in MM patients at relapse (Weinhold et al., 2016). Overexpression of DNA repair machinery could also result in new genomic events especially if nonaccurate pathways like alt-NHEJ are involved (Gourzones-Dmitriev et al., 2013; Viziteu et al., 2017).

## Synthetic Lethality Therapeutic Strategies to Overcome Drug Resistance

DNA repair pathways and other pathways are deregulated in many MM patients to provide adaptive mechanisms and support drug resistance. Targeting DNA repair pathways may potentiate the efficacy of current drugs and overcome drug resistance and represents an opportunity to develop synthetic lethality approaches. Synthetic lethal strategies are currently developed in the context of the DNA damage response with the finding that PARP inhibitors are specifically toxic to HRR defective cancer cells (Bryant et al., 2005; Farmer et al., 2005). Because PI treatment was reported to induce BRCAness in MM cells, (Neri et al., 2011b) this strategy was used for MM cells. Cotreatment of MMCs with bortezomib and PARP1 inhibitor resulted in DSB accumulation and MMC killing (Neri et al., 2011b). Novel compounds targeting DNA repair pathways are being clinically evaluated in patients with cancer to induce synthetic lethality (Shaheen et al., 2011; O’Connor, 2015) (**Table 2**). These inhibitors targeting DNA repair pathways or cell cycle checkpoints (PARP1, DNA-PK, ATM, Ataxia telangiectasia and Rad3 related (ATR), MGMT, APE, CHK1, CHK2) could be useful to target MMCs in combination with DNA-damaging drugs and reverse drug resistance. The choice of which DNA repair inhibitor combine with the different DNA-damaging agents used in MM is related to align the DNA damage induced by the DNA-damaging agent with the DDR repair mechanism. PARP inhibitor was shown to reverse melphalan resistance in human myeloma cell lines, and combination with melphalan has synergistic association to FA and HRR pathways (Xiong et al., 2015). ATR inhibitor also demonstrated significant anti-MM toxicity in the context of MYC-induced replicative stress (Cottini et al., 2015). Interestingly, combination of ATR inhibitor with piperlongumine, a compound increasing ROS levels, exacerbates oncogene-induced DNA damage and shows a synergistic effect (Cottini et al., 2015). CHK1/2 inhibitor AZD7762 was shown to potentiate the effects of melphalan, bendamustine, and doxorubicin in p53-deficient MM cell lines (Landau et al., 2012). Cyclin-dependent kinase (CDK) inhibitors have been identified to impair HRR and sensitize cancer cells to DNA-damaging agents (Jorda et al., 2011; Raghavan et al., 2012). CDK1 and 2 phosphorylate BRCA1 with an important role in the formation of BRCA1 and RAD51 foci and HRR (Ruffner et al., 1999; Johnson et al., 2011). CDK deregulation is a hallmark of MM, and CDK inhibitors are currently under clinical evaluation in MM (Bergsagel et al., 2005; Kumar et al., 2015; Niesvizky et al., 2015). Alagpulinsa et al. (2016) demonstrated that dinaciclib, an inhibitor of CDK, impairs HRR in MM cells and sensitizes them to PARP inhibitor without toxicity to normal B cells. Maes et al. (2014) reported DNA damage induction by DNA methyltransferase inhibitor decitabine in MM cells and stimulation of HRR and NHEJ pathways. Interestingly, combination with HDAC inhibitor or RAD51 inhibitor significantly enhances decitabine-mediated toxicity (Maes et al., 2014). These combinations could be of therapeutic interest in MM with conventional DNA-damaging agents. Several other epigenetic drugs have been shown to enhance the effect of DNA repair inhibitors in other cancers (Orta et al., 2014; Wu et al., 2015). Histone lysine methyltransferase inhibitors could prevent the BRCA1/BARD1 complex at DSBs (Wu et al., 2015).

**Table 2:** DNA damage response inhibitors in clinical development.

Target	Compound
**BER**	
PARP	Olaparib
	Rucaparib
	Niraparib
	Talazoparib
	Veliparib
APE1	Methoxyamine
**HRR**	
ATR	VX-970
	AZD6738
**NHEJ**	
DNA-PKcs	MSC2490484A
	CC-115
**Checkpoint inhibitors**	
CHK1	GDC-0575
	MK-8776
CHK1 and CHK2	LY2606368
	PF-00477736
ATM	AZD0156
WEE1	AZD11775

Achieving success in the clinic with targeted DNA repair inhibitors in combination with DNA-damaging agents will need identification of the MM cell-specific deregulation associated with susceptibility to a DNA repair inhibitor. The GEP-based DNA repair scores recently reported could be of interest in stratifying MM patients and target the addiction of malignant plasma cells to a specific DNA repair pathway. In addition, maximizing the therapeutic window by identifying the correct dose and schedule will be important. In this context, persistence of viable malignant MM cells, within bone marrow, 7 days after high-dose melphalan and ASCT has been reported in two thirds of the patients (Caraux et al., 2012). This therapeutic window could be exploited for residual MM cell eradication before ASCT, taking advantage of new immune therapies presenting favorable toxicity profiles. Furthermore, modulating the redox system would be of therapeutic interest to optimize the use of genotoxic agents in MM (Gourzones et al., 2019).

## Conclusion

Genotoxic agents cause DNA damage that can be recognized by DNA damage response pathways. The accumulation of unrepaired DNA lesions is associated with MM cell apoptosis. MM cells evading DNA damage-induced apoptosis may acquire new genomic alterations that could confer selective advantage and drug resistance. Several mechanisms associated with MMC resistance to genotoxic agents have been described, underlining the MM’s endemic heterogeneous landscape. These findings provide several therapeutic strategies to overcome drug resistance and limit mutagenic effects of genotoxic agents in MM. According to this heterogeneity, adopting precision medicine into clinical practice has the potential to improve MM disease management and treatment. Furthermore, another limitation arises from adverse toxicity on normal cells and tissues, underlining the need to better understand the drug’s mode of action to optimize between efficacy and harmful effects.

## Author Contributions

CG and CB participated in the writing of the paper. JM supervised the writing of the paper.

## Funding

This work was supported by grants from the Institut National du Cancer, INCA, Paris, France, PLBIO15-256, and PLBIO18; 2018-160, ANR (TIE-Skip; 2017-CE15-0024-01), FFRMG, AF3M, Institut Universitaire de France and SIRIC Montpellier (INCa-DGOS-Inserm 6045).

## Conflict of Interest Statement

The authors declare that the research was conducted in the absence of any commercial or financial relationships that could be construed as a potential conflict of interest.
